# Temperature extremes nip invasive macrophyte *Cabomba caroliniana* A. Gray in the bud: potential geographic distributions and risk assessment based on future climate change and anthropogenic influences

**DOI:** 10.3389/fpls.2024.1393663

**Published:** 2024-05-16

**Authors:** Xiaoqing Xian, Yuhan Qi, Haoxiang Zhao, Jingjing Cao, Tao Jia, Nianwan Yang, Fanghao Wan, Philip Weyl, Wan-xue Liu

**Affiliations:** ^1^ State Key Laboratory for Biology of Plant Diseases and Insect Pests, Institute of Plant Protection, Chinese Academy of Agricultural Sciences, Beijing, China; ^2^ Rural Energy and Environment Agency, Ministry of Agriculture and Rural Affairs, Beijing, China; ^3^ Institute of Western Agriculture, Chinese Academy of Agricultural Sciences, Changji, China; ^4^ Centre for Agriculture and Bioscience International (CABI) Centre, Delémont, Switzerland

**Keywords:** bioclimatic variables, anthropogenic activities, freshwater ecosystems, maxent, quarantine

## Abstract

*Cabomba caroliniana* A. Gray, an ornamental submerged plant indigenous to tropical America, has been introduced to numerous countries in Europe, Asia, and Oceania, impacting native aquatic ecosystems. Given this species is a popular aquarium plant and widely traded, there is a high risk of introduction and invasion into other environments. In the current study the potential global geographic distribution of *C. caroliniana* was predicted under the effects of climate change and human influence in an optimised MaxEnt model. The model used rigorously screened occurrence records of *C. caroliniana* from hydro informatic datasets and 20 associated influencing factors. The findings indicate that temperature and human-mediated activities significantly influenced the distribution of *C. caroliniana*. At present, *C. caroliniana* covers an area of approximately 1531×10^4^ km^2^ of appropriate habitat, especially in the south-eastern parts of South, central and North America, Southeast Asia, eastern Australia, and most of Europe. The suitable regions are anticipated to expand under future climate scenarios; however, the dynamics of the changes vary between different extents of climate change. For example, *C. caroliniana* is expected to expand to higher latitudes, following global temperature increases under SSP1–2.6 and SSP2–4.5 scenarios, however, intolerance to temperature extremes may mediate invasion at higher latitudes under future extreme climate scenarios, e.g., SSP5–8.5. Owing to the severe impacts its invasion causes, early warning and stringent border quarantine processes are required to guard against the introduction of *C. caroliniana* especially in the invasion hotspots such as, Peru, Italy, and South Korea.

## 1 Introduction

Plant invasions severely impact freshwater ecosystems, particularly lakes and basins (Stiers et al., 2011; Pan et al., 2023). As the ecosystem with the highest species abundance per unit habitat on Earth (Lozano and Brundu, 2018; Callaghan et al., 2023), freshwater ecosystems may be resilient against plant invasions (Sandvik et al., 2022). However, when an invasion occurs, the rapid population buildup can stress the freshwater ecosystems and severely alter watershed quality and trophic status (Hussner et al., 2017; Haase et al., 2023). Global climate variability and international trade have exacerbated the spread of alien aquatic plants. Climate change leads to fluctuations in environmental elements, such as water temperature, surface level, and nutrient cycling (Bosmans et al., 2022; Rajesh and Rehana, 2022), which may exacerbate the invasion of exotic species and accelerate their spread (Liu et al., 2024). International trade and connectivity between watersheds drive the spread of invasive alien aquatic plants, making it easier for them to be introduced and disperse to new habitats through anthropogenic-mediated activities, such as shipping and aquaculture (Petruzzella et al., 2020). Aquatic invasions may, causing billions of dollars in economic losses to industries such as navigation and fisheries (Lövei et al., 2012), represent a substantial risk to freshwater ecosystems and human economy.

*Cabomba caroliniana* A. Gray (Carolina fanwort; Cabombaceae) is a perennial, herbaceous, submerged aquatic species (CABI Database, 2024) that typically grows in subtropical water at depths of 0.4–1.2 meters (Schooler et al., 2006). It has submerged roots and occasionally floating leaves and flowers, and reproduces sexually by seeds or asexually by leaf shattering (Roberts and Florentine, 2022). *C. caroliniana* is native to the central and eastern United States (Arkansas, Florida, Georgia, North Carolina and South Carolina), Argentina, Brazil, Paraguay, and Uruguay (CABI Database, 2024). It has a high potential for natural spread (Hogsden et al., 2007) owing through vegetative fragmentation promoting downstream dispersal, and typically inhabits nutrient rich ecosystems.

Extensive trade in the aquarium industry and its beautiful ornamental value are significant drivers of its introduction to regions outside its native range (Lima et al., 2014), where it has been documented as having invasive tendencies in Australia, Japan and certain regions of Europe (Bickel and Schooler, 2015). It has been included on the list of major invasive alien plants in Switzerland (2014), China (2016) and Chile (2019). More noteworthy is the fact that *C. caroliniana* was listed as a union species in Europe in 2016 (EPPO Global Database, 2024).

*C. caroliniana* commonly invades habitats with low species diversity because it has a wider ecological niche (Bickel, 2015) than native species, and therefore poses a serious threat to them. According to Zhang et al. (2003), the introduction of *C. caroliniana* to Asia has been associated with a significant threat to *Ottelia alismoides* (L.) Pers., a species that was once widespread but became rarely observed thereafter. The invasion of *C. caroliniana* not only clogs freshwater ecosystems, but also leads to a drastic decline in the biodiversity of indigenous aquatic plants (Zhang et al., 2003).

Species distribution models (SDMs) are based on the theory of ecological niches and utilise known population distribution sites and associated environmental factors, such as climate, soil, topography and ultraviolet light, to project the magnitude of change in the environmental space suitable for species survival (Suggitt et al., 2023). Maximum Entropy (MaxEnt) model is one of the most widely applied SDMs, which is based on machine learning with maximum entropy utilising a species’ known geographic distribution and associated environmental factors to infer its ecological requirements to further project their potential geographic distributions (PGDs) in the study area (Karuppaiah et al., 2023). Previous studies have shown that if all acquired environmental variables are generalised and involved in the modelling, this increases the complexity of the model, greatly reduces the ability to transfer species, and limits model performance (Wang et al., 2023). Therefore, the optimised MaxEnt model has the significant advantage of preventing overfitting. In recent years, optimised MaxEnt models used to predict the PGDs of alien aquatic plants have become more refined (Kariyawasam et al., 2021; Zhang et al., 2021; Yasuno, 2022). However, these studies generally suffer from the shortcoming that few of the acquired species distribution records are extracted by overlaying them with sophisticated river network data, resulting in incomplete predicted PGDs for freshwater ecosystems. In this study, we further refined and processed the occurrence records with precision, effectively improving the accuracy of the rational prediction of habitats and PGDs during the modelling process.

The invasion of *C. caroliniana* has brought strong competition and biodiversity loss to the native flora of freshwater ecosystems, negatively affecting the ecology of river networks. The study of climate change- and trade-driven PGDs can provide reference values for early monitoring and warning systems. Here, we 1) screened the current global occurrence records of *C. caroliniana*, 2) optimised the traditional MaxEnt model and obtained optimal parameter combinations, 3) ranked the influencing factors driving the spread of *C. caroliniana*, 4) predicted the global PGDs of *C. caroliniana* under existing and future climate scenarios, and 5) discussed the spatial variation in PGDs under future climate change. Our study provides guidance for assessing the spread of *C. caroliniana* and for protecting the ecological integrity of freshwater ecosystems.

## 2 Data and methods

### 2.1 Occurrence record sources

Information on the occurrence records of *C. caroliniana* was obtained in two ways: a search of reported locations through the related published literature in the Web of Science (WOS), and obtaining the corresponding latitude and longitude coordinates using the Baidu Coordinate Gathering System (BCGS) (https://api.map.baidu.com/lbsapi/getpoint/). Secondly, latitude and longitude information were collected from occurrence records and specimen collection records in the Chinese Virtual Herbarium (CVH), Centre for Agriculture and Bioscience International (CABI) (CABI Database, 2024), European and Mediterranean Plant Protection Organization (EPPO) (EPPO Global Database, 2024) and the Global Biodiversity Information Facility (GBIF) (GBIF.org, 2024). Our investigation yielded a comprehensive dataset comprising 3680 initial occurrence records of *C. caroliniana* sourced from various channels.

### 2.2 Factors influencing the distribution of *C. caroliniana*

The factors influencing *C. caroliniana* are mainly bioclimatic and anthropogenic (**Supplementary Table S1**). Nineteen current bioclimatic factors (bio1–bio19) were downloaded from the World Climate Database at a resolution of 5 arcmin. Future bioclimatic factors were obtained from the WorldClim database (Fick and Hijmans, 2017). Three shared socioeconomic pathways (SSPs), namely SSP1–2.6, SSP2–4.5 and SSP5–8.5, were employed for the 2030s and the 2050s. These pathways represent future scenarios with varying levels of carbon dioxide (CO_2_) concentrations based on the Beijing Climate Center Climate System Model (BCC-CSM2-MR). SSP1–2.6, SSP2–4.5 and SSP5–8.5 signified low, medium and high CO_2_ concentrations, respectively (Wu et al., 2019). The Human Influence Index was obtained from the Socioeconomic Data and Applications Center (SEDAC) of NASA. In cases where two factors exhibited Pearson correlations of |r| > 0.8, only the factor with the highest correlation was chosen for modelling.

### 2.3 Model optimization and precision

Calibration of feature combinations (FCs) and a regularisation multiplier (RM) can appreciably improve the prediction precision of a MaxEnt model (Javidan et al., 2021; Betts et al., 2022). FCs are set with five basic parameters (linear–L, quadratic–Q, hinge–H, product–P and threshold–T), while RM is incremented at intervals of 0.5 from 0.5 to a maximum of 4. A total of 48 different combinations are available. We utilised the ENMeval package in R v 4.2.1 software (R Development Core Team) to generate candidate models (Cobos et al., 2019). Finally, we selected the model with a significant delta value as the optimal model, with an AICc value of 0. The accuracy of the model was examined using the area enclosed by the area of under receiver operating characteristic (ROC) curve (AUC).

### 2.4 Classification of PGDs at different risk levels

The ASCII file output from the MaxEnt model was converted to raster format (tif) and the corresponding raster value was the potential fitness probability (P) of *C. caroliniana* in the area to be predicted. Based on the maximum test sensitivity and specificity cloglog threshold, the reclassification command in the spatial analysis tool of ArcGIS 10.7 was utilised to classify PGDs with different risk levels into four classes: the unsuitable habitats (0<P ≤ 0.22), low suitability habitats (0.22<P ≤ 0.4), moderate suitability habitats (0.4<P ≤ 0.6), and high suitability habitats (0.6<P ≤ 1).

### 2.5 Acquisition of centroids

A centroid indicator was used to characterise shifts in the geospatial distribution of a species. The raster figures of PGDs under specific climate scenarios in various periods were vectorised first, and then ArcGIS was used to calculate the position of the centroids (the geographic centre of the PGDs) of *C. caroliniana* on each continent and to compare the geospatial variations and direction of the shift of *C. caroliniana* on diverse continents of the world over time (Zhang et al., 2023).

## 3 Results

### 3.1 Current global distribution of *C. caroliniana*

**Figure 1:**
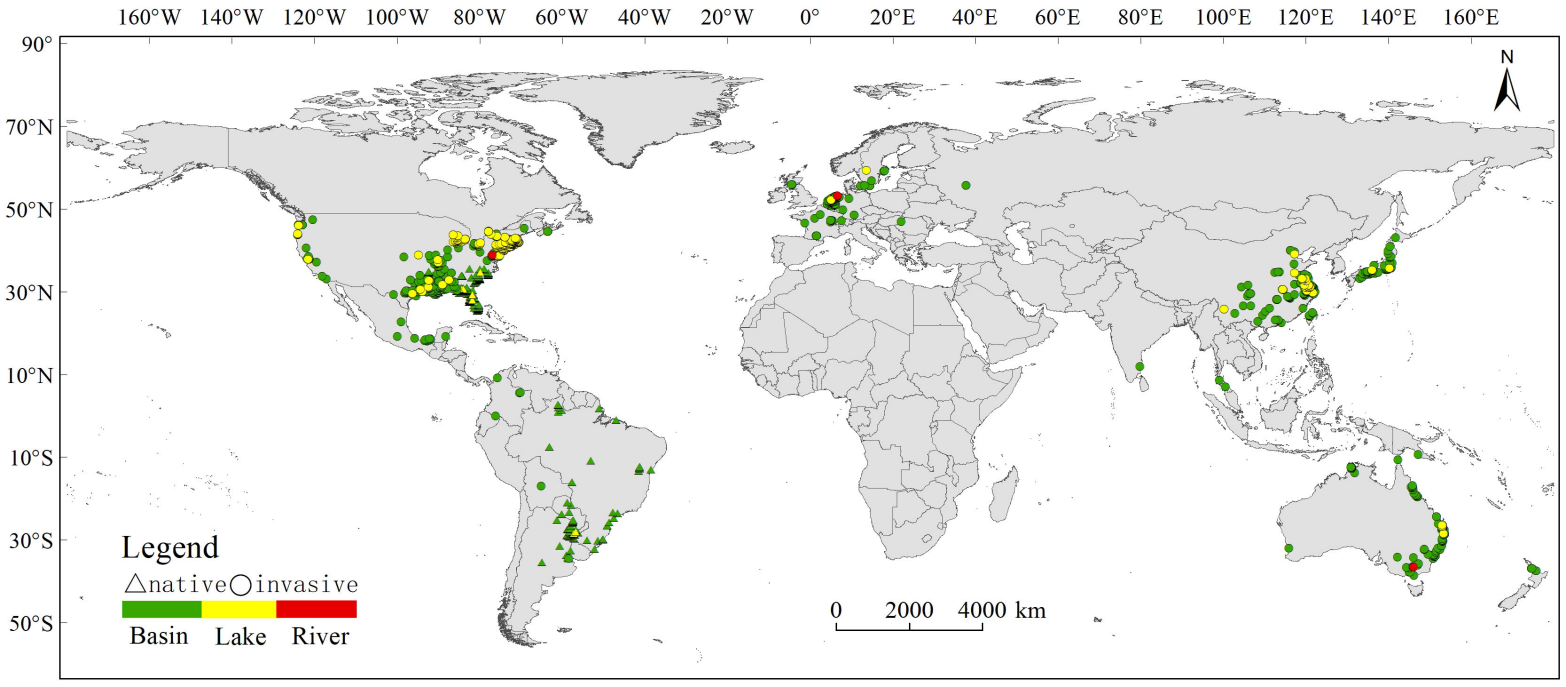
Current distribution of *Cabomba caroliniana* in different countries and water types (basin/lake/river).

After meticulous filtration to exclude instances related to indoor cultivation and purchases, our refined dataset consisted of 3553 occurrence records, ensuring data precision. To enhance the robustness of our dataset for subsequent analyses, we meticulously overlaid the occurrence records with global hydrological data sourced from the HydroSHEDS dataset, including HydroBASINS, HydroRIVERS, and HydroLAKES. This stringent process yielded a final dataset of 3155 validated occurrence records (**Figure 1**), categorized into basin, lake, or river contexts. Overall, *C. caroliniana* was currently invasive worldwide except from Africa and Antarctica, and the basin was the dominant water body type.

### 3.2 Optimized models

The MaxEnt model was optimised to predict potential geographic distributions of *C. caroliniana*. The base data included 3155 occurrence records of *C. caroliniana* and 20 influencing factors. The results showed that setting FCs to LQHP and RM to 0.5 were the best parameters for this simulation. For this parameter, 10 simulation repetitions were performed which obtained an average AUC value of 0.933. We predicted the average AUC values under future climate change scenarios to be 0.932, 0.935, 0.933, 0.934, 0.933, and 0.932, respectively. The optimised MaxEnt model showed good performance in terms of the precision of the prediction results of the PGDs of *C. caroliniana* (**Supplementary Figure S1**).

### 3.3 Significance of influencing factors

**Figure 2:**
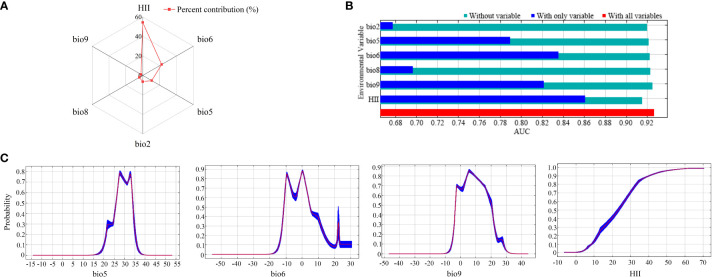
**(A)** Percent contribution and **(B)** jackknife results for the six influential factors affecting the presence probability of *Cabomba caroliniana*, and **(C)** response curves of the four most influential factors.

Among all 20 candidate influences (**Supplementary Table S1**), the factors that showed the most significant relationship with PGDs of *C. caroliniana* were the temperature elements (bio2, bio5, bio6, bio8 and bio9) and the Human Influence Index (HII). In the model-fitting process, the contribution rate depicted the hierarchy of significance among the factors influencing the PGDs of *C. caroliniana* (**Figure 2A**). The pinnacle three factors with the highest contributions were HII (54.3%), the minimum temperature of the coldest month (bio6, 22.4%) and the maximum temperature of the warmest month (bio5, 10.6%), with a cumulative contribution of 87.3%. Jackknife test results showed that the three factors governing the PGDs of *C. caroliniana* were HII, bio6 and the mean temperature of the driest quarter (bio9), indicating that these factors were significantly more influential than the others (**Figure 2B**).

Commonly, a fitness probability greater than 0.5 is considered to be a suitable syndrome of such environmental conditions for the growth of alien plants. Therefore, considering the significant environmental variables of the response curve of *C. caroliniana*, the max temperature of warmest month suitable for *C. caroliniana* growth ranged from 25°C to 34°C, minimum temperature of coldest month ranged from -11°C to 5°C, mean temperature of driest quarter ranged from -3°C to 20°C, and the Human Influence Index was greater than 22 (**Figure 2C**).

### 3.4 PGDs globally under current and future climate scenarios

**Figure 3:**
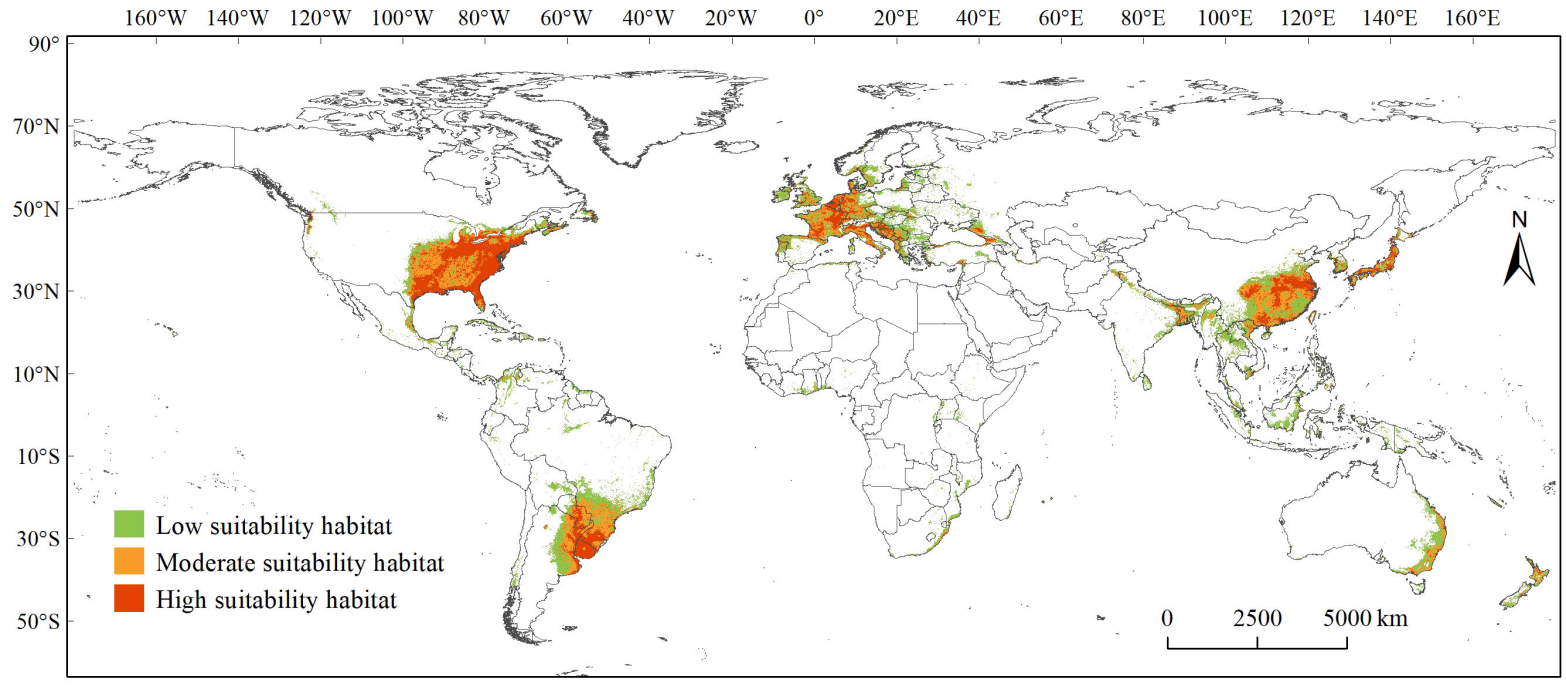
PGDs of *Cabomba caroliniana* globally under the current climate scenario.

**Figure 4:**
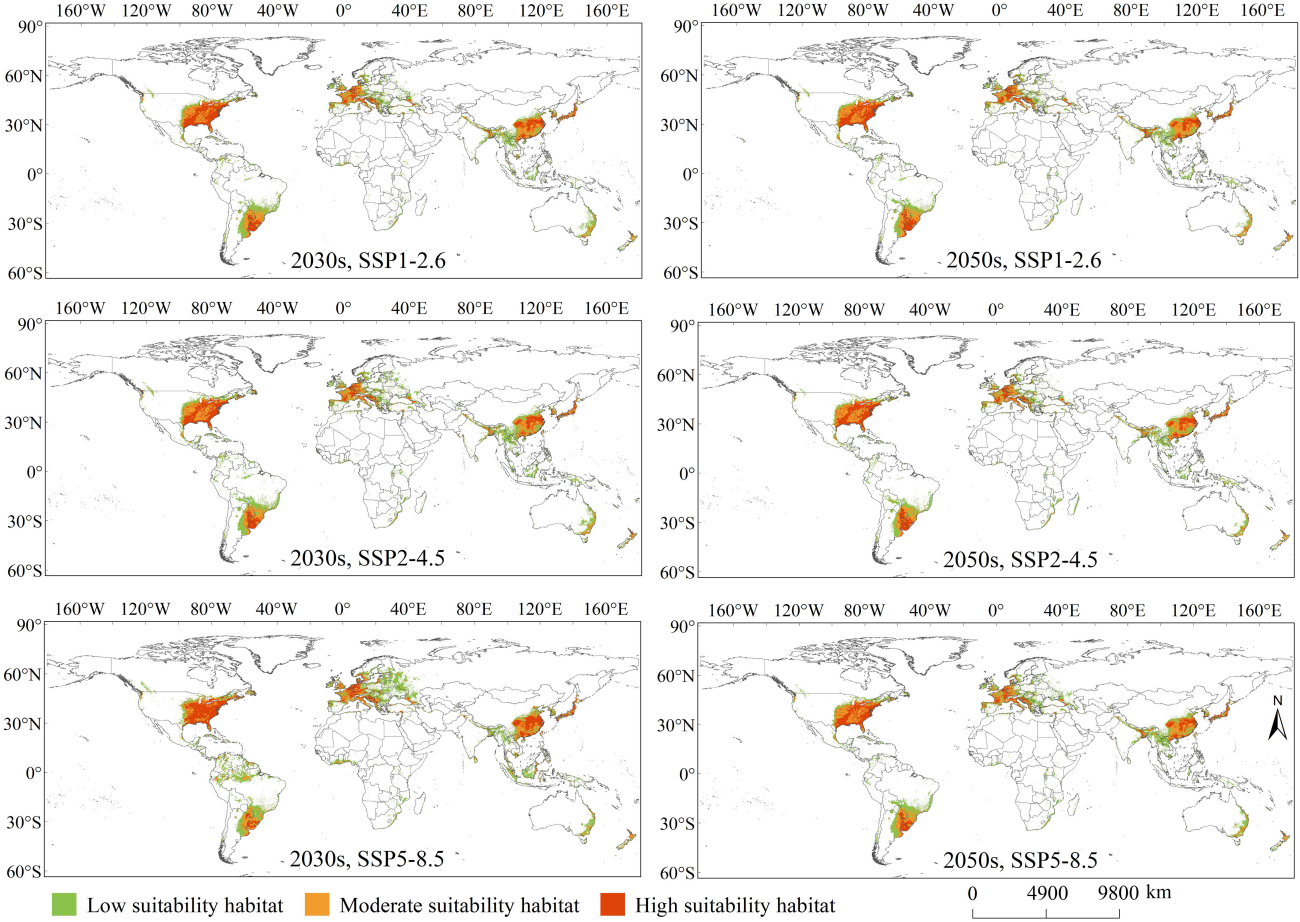
PGDs of *Cabomba caroliniana* globally under future climate scenarios.

Overall, the PGDs of *C. caroliniana* under the current and future climate scenarios were mainly distributed in south-eastern South and North America, Central America, eastern and Southeast Asia, eastern Australia, and most of Europe (**Figures 3**, **4**). The geographic pattern of global PGDs predicted under future climate scenarios (**Figure 4**) did not change considerably compared to the current climate (**Figure 3**), but increased in area to varying degrees. Under SSP5–8.5, the area of PGDs of *C. caroliniana* reached a maximum in the 2030s and 2050s, and in particular the area of PGDs in the 2030s is expected to peak in the future.

Specifically, under the existing climate scenario (**Figure 3**), the high suitability habitat area was approximately 399 ×10^4^ km^2^, accounting for approximately 26% of PGDs globally, mainly distributed in South America, North America, western Europe, eastern Asia and Australia. The specific countries have been collated in **Supplementary Table S2**. The moderate suitability habitat area was approximately 453 ×10^4^ km^2^, accounting for approximately 30% of PGDs globally. In contrast, the low suitability habitat area was approximately 679 ×10^4^ km^2^, accounting for approximately 44% of PGDs globally, and are scattered distributions on all six continents except Antarctica (**Supplementary Table S2**).

The geographic distribution pattern of PGDs of *C. caroliniana* under future climate scenarios was generally consistent with the current distribution pattern (**Figure 4**). For the SSP1–2.6 scenario in the 2030s, the high suitability habitat area was approximately 420 ×10^4^ km^2^ (26% of PGDs globally), moderate suitability habitat area was approximately 454×10^4^ km^2^ (28%), and low suitability habitat area was approximately 730×10^4^ km^2^ (46%). Under 2050s and SSP1–2.6, the high suitability habitat area was approximately 427×10^4^ km^2^ (28%), moderate suitability habitat area was approximately 462×10^4^ km^2^ (29%), and low suitability habitat area was approximately 661×10^4^ km^2^ (43%).

For the SSP2–4.5 scenario in 2030, the high suitability habitat area was approximately 405×10^4^ km^2^ (24%), moderate suitability habitat area was approximately 460×10^4^ km^2^ (27%), and the low suitability habitat area was approximately 833×10^4^ km^2^ (49%). Under 2050s and SSP2–4.5, the high suitability habitat area was approximately 414×10^4^ km^2^ (27%), moderate suitability habitat area was approximately 460×10^4^ km^2^ (30%), and the low suitability habitat area was approximately 662×10^4^ km^2^ (43%).

For scenario SSP5–8.5 in the 2030s, the high suitability habitat area was approximately 499×10^4^ km^2^ (26%), moderate suitability habitat area was approximately 494×10^4^ km^2^ (26%), and the low suitability habitat area was approximately 937×10^4^ km^2^ (48%). Lastly, for SSP5–8.5 in the 2050s, the high suitability habitat area was approximately 399×10^4^ km^2^ (25%), the moderate suitability habitat area was approximately 447×10^4^ km^2^ (28%), and the low suitability habitat area was approximately 737×10^4^ km^2^ (47%).

### 3.5 Spatial variations of PGDs globally

**Figure 5:**
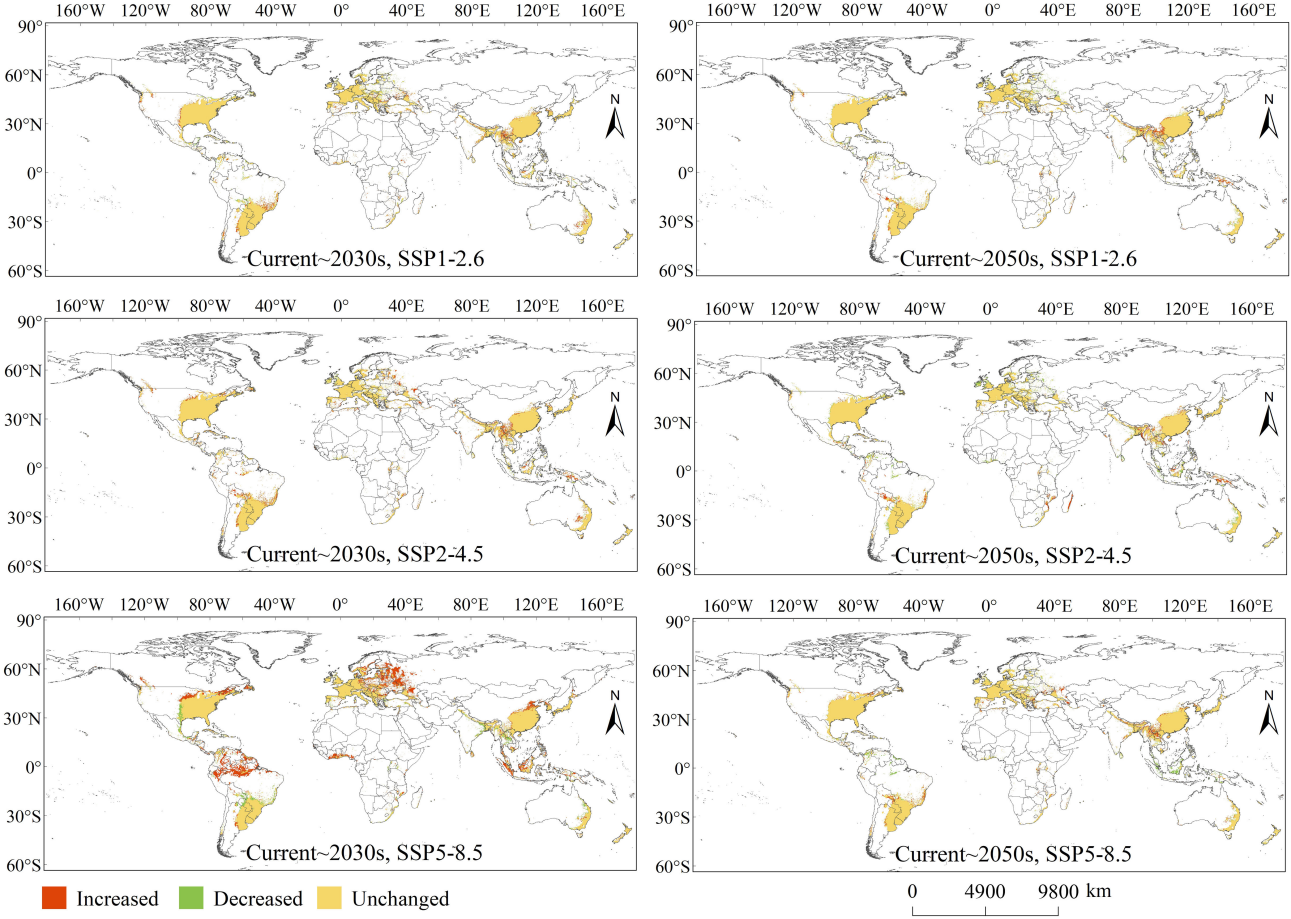
Variations of PGDs of *Cabomba caroliniana* globally under future climate condition.

Identifying and visualising the spatial variation of PGDs globally helps reflect the magnitude of impacts from different climate scenarios. Compared with the existing climate scenario, the spatial variation of PGDs globally for *C. caroliniana* under different future climate scenarios were classified as “Increased”, “Decreased” and “Unchanged” (**Figure 5**). Areas of “Increased” demonstrate that under future climatic situations the environmental variables become more conducive to the survival of *C. caroliniana*; areas of “Decreased” indicate that under future climatic situations, the habitat will no longer be appropriate for *C. caroliniana* and the habitat will disappear.

In the 2030s, future increases in PGDs were especially located in the central and northern United States, northern South America, north-eastern Europe, western Africa, south-western and northern China, and Indonesia, with more significant increases under SSP5–8.5. Future decreases in PGDs were especially located in the central United States, south-eastern Mexico, central South America, central Europe, South Asia, Southeast Asia, and eastern Australia, with more salient decreases under SSP5–8.5. In the 2050s, future increases in PGDs were especially in central Bolivia, Argentina, Brazil, southern Russia, south-western China, South Asia, and Southeast Asia, with more significant increases under SSP5–8.5. Future decreases in PGDs were especially located in northern Central and South America, central Europe, Southeast Asia, and eastern Australia, with more significant decreases under SSP5–8.5. Notably, in the 2030s, the SSP5–8.5 scenario had the most significant variation in the extent of global PGDs.

Specifically, compared to the current climate scenario, under SSP1–2.6, the PGDs area approximately increased by 149×10^4^ km^2^, decreased by 74×10^4^ km^2^ and remained unchanged at 1454×10^4^ km^2^ in the 2030s, and then increased by 119×10^4^ km^2^, decreased by 89×10^4^ km^2^ and remained unchanged at 1437×10^4^ km^2^ in the 2050s. Under SSP2–4.5, the PGDs area increased by 210×10^4^ km^2^, decreased by 45×10^4^ km^2^ and remained unchanged at 1486×10^4^ km^2^ in the 2030s, and then increased by 119×10^4^ km^2^, decreased by 90×10^4^ km^2^ and remained unchanged at 1385×10^4^ km^2^ in the 2050s. Under SSP5–8.5, the PGDs area increased by 536×10^4^ km^2^, decreased by 134×10^4^ km^2^ and remained unchanged at 1391×10^4^ km^2^ in the 2030s, and then increased by 149×10^4^ km^2^, decreased by 104×10^4^ km^2^ and remained unchanged at 1433×10^4^ km^2^ in the 2050s.

**Figure 6:**
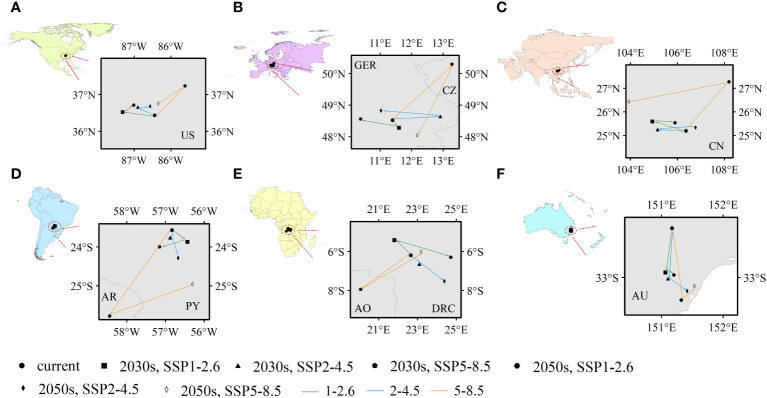
**(A–F)** Future centroid shifts of *Cabomba caroliniana* on various continents under different climate scenarios (US, the United States; GER, Germany; CZ, Czechia; CN, China; AR, Argentina; PY, Paraguay; AO, Angola; DRC, Republic of the Congo; AU, Australia).

Climate-mediated centroid variations in PGDs were further analysed that could reflect spatial variations, mainly latitudinal and longitudinal, in potentially suitable habitats for *C. caroliniana* (**Figure 6**). For the six continents (except Antarctica), the geographic centroids of the PGDs of *C. caroliniana* were obtained using ArcGIS for each climate scenario (**Supplementary Table S3**), and some trends were derived. In terms of shifting trends, the centroid variations in PGDs under SSP5–8.5 were inconsistent with or even opposite to those under the remaining two climate scenarios. Under the SSP1–2.6 and SSP2–4.5 scenarios, the PGDs of *C. caroliniana* consistently shifted to higher latitudes, whereas under SSP5–8.5, they shifted to higher and then lower latitudes. In terms of the scope of shifting, the PGDs in North America, Asia, and Oceania did not shift beyond the administrative boundaries of one country–the United States, China, or Australia–although spatial shifts were observed. In contrast, under the SSP1–2.6 and SSP2–4.5 scenarios, PGDs in Europe, South America and Africa were not shifted beyond the administrative boundaries of more than one country, i.e. Germany, Paraguay and the Democratic Republic of the Congo, respectively. However, under the SSP5–8.5 scenario, they shifted to Czechia, Argentina and Angola, respectively.

## 4 Discussion

*C. caroliniana* has been described as a dominant competitive alien plant in water bodies of Europe and China owing to its rapid reproduction and competitive ability (EPPO Global Database, 2024). However, to date, this issue has not received much attention with most research targeting life history (Lima et al., 2014; Roberts and Florentine, 2022) and localised suitable habitats (Fan et al., 2019). Here we provide, for the first time, a reference for potential trends in the global geographic distribution of *C. caroliniana* under the influence of climate variability and anthropogenic activities.

### 4.1 Effects of influencing factors on PGDs

Temperature is one of the maximum critical elements influencing plant metabolism, physiological activities and developmental status. Extreme temperatures may lead to oxidative stress and disruption of cell membranes in aquatic plants (França et al., 2007; Zhang et al., 2022), which in turn influence their potential geographic distributions. The extremes of three temperature factors appeared to synergistically affect the PGDs of *C. caroliniana* and exert the most influence in determining habitat suitability – maximum temperature of the warmest month, minimum temperature of the coldest month and mean temperature of the driest quarter. Suitability of the environment for *C. caroliniana* tended to zero when the max temperature of the warmest month was below 20°C or above 36°C, minimum temperature of coldest month was below -13°C or above 18°C, and mean temperature of driest quarter was below -5°C or above 29°C. Our findings are supported by previous studies that *C. caroliniana* typically thrives in acidic, sluggish water with an optimal temperature range for growth being 13–27°C (Bickel, 2017). Although extreme temperatures appear to be a limiting factor, being a submerged species, it is possible that the water provides a buffer to the extreme temperatures allowing *C. caroliniana* to invade regions with large fluctuations in high and low temperatures such as Europe.

In addition to temperature, anthropogenic activities such as the aquatic plant trade and navigation have been identified as another important factor mediating the expansion of PGDs of *C. caroliniana*. Several points of invasion have been linked to trade (Hogsden et al., 2007) and transport (Bickel, 2015; Sardain et al., 2019). *C. caroliniana* is capable of surviving long periods of desiccation and has the ability to spread to new regions through the movement of boats and recreational equipment despite long periods of desiccation (Bickel, 2015).

### 4.2 Variations of PGDs globally

Predicting the potential global distribution can identify areas or potential hotspots for the invasion of *C. caroliniana*, and help identify areas suitable for *C. caroliniana* establishment and growth. *C. caroliniana* has widespread PGDs in areas other than those with known occurrences, highlighting the risk to countries and regions, such as the United States, Northern Europe, Russia, and Japan. The future climate change predictions of SSP5–8.5 scenario in the 2030s had the largest PGDs expansion, suggesting that this scenario is the most conducive to the habitat suitability of *C. caroliniana*. The predicted suitable areas contract under 2050s predictions suggesting a probable attainment of a temperature threshold for *C. caroliniana* compounded by the potential proliferation of phytoplankton may reduce light in the water column, restricting the growth of submerged aquatic plants such as *C. caroliniana* (Kosten et al., 2011; Arthaud et al., 2012; Asaeda and Rashid, 2017). In addition, studies have demonstrated that the increase in atmospheric CO_2_ concentrations through anthropogenic activities (Ciais et al., 2013; Cheng et al., 2022; Xu et al., 2023) can have both direct and indirect effects on the submerged plant *C. caroliniana* (Gamage et al., 2018). However, the positive effects of increased atmospheric CO_2_ concentrations on *C. caroliniana* diminish with increasing concentrations suggesting that the benefits *C. caroliniana* obtains from increased CO_2_ cannot be sustained long term (Deng et al., 2013; Burnell et al., 2014; Cao and Ruan, 2015; van Kempen et al., 2016).

As global carbon and greenhouse gas emissions increase, the range of invasive alien plants is likely to shift, especially the establishment of invasive plants at higher latitudes (Ouyang et al., 2021; Zhao et al., 2021; Zhang et al., 2023). However, our findings suggest that there are limitations to this conclusion. Under the SSP1–2.6 and SSP2–4.5 scenarios, the relatively slow increase in global greenhouse gas concentrations, a less extreme climate change allows the expansion of *C. caroliniana* to higher latitudes. However, under more extreme climate change scenario, SSP5–8.5, with more rapid global carbon emissions, the magnitude of climate change increases with more dramatic temperature changes are likely to limit the expansion of *C. caroliniana* at higher latitudes. But it may facilitate expansion of suitable habitats at migration to lower latitudes (Jia et al., 2023). Overall, global climate change will result in increasing PGDs of *C. caroliniana* globally, however, intolerance to extreme temperatures may mediate the loss of high-latitude habitats to *C. caroliniana* under some future climate change scenarios.

### 4.3 Early warning and management efforts

For countries where climatic conditions match the preferences of *C. caroliniana* and where it has not yet been found the risk of introduction an establishment can be mitigated by tightened border security. However, many potential difficulties exist in that endeavour. For instance, *C. caroliniana* may be introduced into a country through multiple means, including illegal trade, water drift and migratory animals, and its path of spread is difficult to fully predict and control. In the face of these challenges, there is a need to establish a sound early warning system and develop integrated management measures to deal with them. Firstly, early warning systems can help to monitor and detect the spread of aquatic invasive plants such as *C. caroliniana* in a timely manner. For example, by monitoring changes in the vegetation cover of waters such as lakes, rivers and watercourses, signs of invasive plants can be detected and identified in a timely manner so that targeted management measures can be taken. Secondly, with the help of early warning systems, data on the distribution, growth status and ecological impact of invasive plants are collected and analysed, and management measures (one measure for one species) are developed in a targeted manner. For example, different management methods, such as physical barrier, biological control or chemical containment, may be adopted in specific areas, with choices based on the characteristics of the invasive plants and the conditions of their habitats, in order to minimize ecological damage. Finally, as aquatic invasive plants often crossing national borders, transnational cooperation and joint efforts are needed to dress them. It is recommended that an international early warning network and information exchange mechanism be established to ensure the timely uploading and sharing of invasive plant monitoring data and management experience, and that international cooperation and coordination be strengthened in order to jointly deal with the threat posed by invasive plants to ecosystems and economies.

However, the implementation process will face many unknowns and obstacles. Firstly, the establishment of monitoring and early warning systems requires a considerable amount of financial investment and technical support. It involves costs and technology for setting up monitoring stations, collecting data, and conducting data analysis and processing. Secondly, the management of aquatic invasive plants involves multiple stakeholders and there may be a lack of clarity in management responsibilities and authority. There is the possibility of information asymmetry, for example, between different agencies or departments, resulting in impediments to the implementation of management measures. In addition, the spread of aquatic invasive plants is spatially-temporally complicated and potentially affected by both natural and anthropogenic factors, which are bound to slip through the cracks of even a well-developed early warning system. In conclusion, subsequent researchers should focus on joint efforts in prevention, ecological research, management and governance, international co-operation and promotion of public participation to effectively control invasive alien aquatic plants and protect the health and stability of aquatic ecosystems.

### 4.4 Limitations

Our study used bioclimatic variables to predict the potential geographic distribution of invasive plants in freshwater ecosystems with good prediction precision, but as well, we recognised some model limitations. Firstly, bioclimatic factors, while providing vital environmental information on plant growth and distribution, do not fully take into account the hydrological conditions and nutrient status of water bodies that are specific to freshwater ecosystems. These factors may interact with bioclimatic factors, leading to increased uncertainty about the range of potential geographic distributions. Another major limitation is our failure to adequately account for the effects of resource availability on the distribution of invasive plants. The impact of resource availability, particularly eutrophication, on macroalgal communities can be critical, but due to limitations of the current Maxent model, we were unable to integrate this factor into our analyses. This means that the disturbance of invasive plant distributions by resource alterations may not be fully captured by our models, which may affect the integrity of the model.

Nonetheless, our findings provide important insights into understanding of the invasive plant distributions in freshwater ecosystems. We emphasise that the aim of this study was to provide an initial predictive framework to help guide practices of invasive weed management. Subsequent studies could further consider factors such as resource availability and incorporate other modelling approaches to improve prediction accuracy.

## 5 Conclusions

The predictions based on the MaxENT modelling suggest that temperature extremes will likely influence the distribution and potential spread of *C. caroliniana*. Under the milder climate change predictions of SSP1–2.6 and SSP2–4.5, it is likely that *C. caroliniana* will expand to higher latitudes in the future (2030s and 2050s), but this may be restricted by its intolerance to excessive temperatures in these regions under more extreme future extreme climate scenarios such as SSP5–8.5. Given the impacts pf *C. caroliniana*, early warning and stringent quarantine processes are needed to prevent its uncontrolled spread in current global invasion hotspots such as East Asia, Oceania, and Europe, as well as in suitable countries that it has not yet colonized, such as Peru, Italy, and South Korea. In the regions where it is already established, the predictions may be used as a guide where to best implement management practices for the most efficient use of limited resources.

## Data availability statement

The raw data supporting the conclusions of this article will be made available by the authors, without undue reservation.

## Author contributions

XX: Conceptualization, Investigation, Project administration, Resources, Supervision, Validation, Visualization, Writing – review & editing. YQ: Conceptualization, Data curation, Formal analysis, Investigation, Methodology, Software, Writing – original draft. HZ: Conceptualization, Supervision, Validation, Writing – review & editing. JC: Data curation, Formal analysis, Investigation, Writing – original draft. TJ: Conceptualization, Formal analysis, Investigation, Project administration, Validation, Writing – review & editing. NY: Conceptualization, Formal analysis, Investigation, Validation, Writing – review & editing. F-HW: Investigation, Supervision, Validation, Writing – review & editing. PW: Conceptualization, Funding acquisition, Project administration, Supervision, Validation, Visualization, Writing – review & editing. WL: Conceptualization, Funding acquisition, Project administration, Supervision, Validation, Visualization, Writing – review & editing.
