# Supplementary material for: Temperature extremes nip invasive macrophyte Cabomba caroliniana A. Gray in the bud: potential geographic distributions and risk assessment based on future climate change and anthropogenic influences
Source: Front Plant Sci. 2024 May 16;15:1393663. doi: 10.3389/fpls.2024.1393663 (PMC11137650; doi:10.3389/fpls.2024.1393663)
Supplement: Supplementary file 1 [file DataSheet_1.docx]

**Supplementary File**

Tab. S1 the 20 influencing factors and their categories, abbreviations, and full names

| Category | Abbreviation | Full name |
| --- | --- | --- |
| bioclimatic | bio1 | Annual Mean Temperature |
|  | bio2 | Mean Diurnal Range (Mean of monthly (max temp - min temp)) |
|  | bio3 | Isothermality (bio2/bio7) (×100) |
|  | bio4 | Temperature Seasonality (standard deviation ×100) |
|  | bio5 | Max Temperature of Warmest Month |
|  | bio6 | Min Temperature of Coldest Month |
|  | bio7 | Temperature Annual Range (bio5-bio6) |
|  | bio8 | Mean Temperature of Wettest Quarter |
|  | bio9 | Mean Temperature of Driest Quarter |
|  | bio10 | Mean Temperature of Warmest Quarter |
|  | bio11 | Mean Temperature of Coldest Quarter |
|  | bio12 | Annual Precipitation |
|  | bio13 | Precipitation of Wettest Month |
|  | bio14 | Precipitation of Driest Month |
|  | bio15 | Precipitation Seasonality (Coefficient of Variation) |
|  | bio16 | Precipitation of Wettest Quarter |
|  | bio17 | Precipitation of Driest Quarter |
|  | bio18 | Precipitation of Warmest Quarter |
|  | bio19 | Precipitation of Coldest Quarter |
| anthropogenic | HII | Human Influence Index |

Tab. S2 Countries and regions with distribution of *Cabomba caroliniana* currently under different potential geographic distribution categories

| Categories | Countries and regions |
| --- | --- |
| low suitability habitats | Central United States; Central and South America; most of Europe; Cote d’ Ivoire; Ghana; Benin; Uganda; Kenya; Mozambique; South Africa; India; Nepal; Bangladesh; Myanmar; Thailand; Laos; Vietnam; Philippines; Indonesia; South-eastern China; Korea; Japan; eastern Australia and New Zealand |
| moderate suitability habitats | Central and eastern United States; North-eastern Argentina; Southern Brazil; France; Netherlands; Germany; Italy; Northern Switzerland; Slovenia; Serbia; Albania; South-western Russia; Georgia; Bangladesh; Myanmar; Vietnam; South-eastern China; Japan; Eastern Australia and northern New Zealand |
| high suitability habitats | Eastern and southern United States; Paraguay; Uruguay; Southern Brazil; United Kingdom; France; Belgium; Netherlands; Germany; Northern Switzerland; Italy; Croatia; South-western Russia; Bangladesh; South-eastern China; Japan; Northern New Zealand |

Tab. S3 Centroids of potential geographic distribution of *Cabomba caroliniana* on continents (except Antarctica) under different climate scenarios

| Continent | Scenario | Longitude (°) | Latitude (°) |
| --- | --- | --- | --- |
| Africa | Current climate | 22.66 | -6.20 |
|  | 2030s, SSP1-2.6 | 21.82 | -5.42 |
|  | 2030s, SSP2-4.5 | 23.10 | -6.64 |
|  | 2030s, SSP5-8.5 | 20.08 | -7.94 |
|  | 2050s, SSP1-2.6 | 24.72 | -6.30 |
|  | 2050s, SSP2-4.5 | 24.39 | -7.52 |
|  | 2050s, SSP5-8.5 | 23.19 | -6.01 |
| Asia | Current climate | 106.37 | 25.19 |
|  | 2030s, SSP1-2.6 | 104.94 | 25.59 |
|  | 2030s, SSP2-4.5 | 105.16 | 25.25 |
|  | 2030s, SSP5-8.5 | 108.20 | 27.28 |
|  | 2050s, SSP1-2.6 | 105.90 | 25.53 |
|  | 2050s, SSP2-4.5 | 106.78 | 25.34 |
|  | 2050s, SSP5-8.5 | 103.94 | 26.44 |
| Europe | Current climate | 11.40 | 48.52 |
|  | 2030s, SSP1-2.6 | 11.60 | 48.28 |
|  | 2030s, SSP2-4.5 | 12.58 | 48.56 |
|  | 2030s, SSP5-8.5 | 13.28 | 50.30 |
|  | 2050s, SSP1-2.6 | 10.38 | 48.56 |
|  | 2050s, SSP2-4.5 | 11.03 | 48.83 |
|  | 2050s, SSP5-8.5 | 12.18 | 48.05 |
| North America | Current climate | -86.44 | 36.43 |
|  | 2030s, SSP1-2.6 | -87.31 | 36.52 |
|  | 2030s, SSP2-4.5 | -86.90 | 36.66 |
|  | 2030s, SSP5-8.5 | -85.61 | 37.24 |
|  | 2050s, SSP1-2.6 | -87.01 | 36.71 |
|  | 2050s, SSP2-4.5 | -86.56 | 36.68 |
|  | 2050s, SSP5-8.5 | -86.34 | 36.76 |
| Oceania | Current climate | 153.45 | -32.66 |
|  | 2030s, SSP1-2.6 | 152.69 | -32.85 |
|  | 2030s, SSP2-4.5 | 152.26 | -32.60 |
|  | 2030s, SSP5-8.5 | 154.93 | -33.32 |
|  | 2050s, SSP1-2.6 | 152.71 | -32.89 |
|  | 2050s, SSP2-4.5 | 152.70 | -33.29 |
|  | 2050s, SSP5-8.5 | 152.55 | -33.07 |
| South America | Current climate | -56.83 | -23.57 |
|  | 2030s, SSP1-2.6 | -56.43 | -23.87 |
|  | 2030s, SSP2-4.5 | -56.88 | -23.76 |
|  | 2030s, SSP5-8.5 | -58.44 | -25.77 |
|  | 2050s, SSP1-2.6 | -57.15 | -23.99 |
|  | 2050s, SSP2-4.5 | -56.67 | -24.18 |
|  | 2050s, SSP5-8.5 | -56.30 | -24.75 |

^*^ ‘-’ indicates west longitude or south latitude.


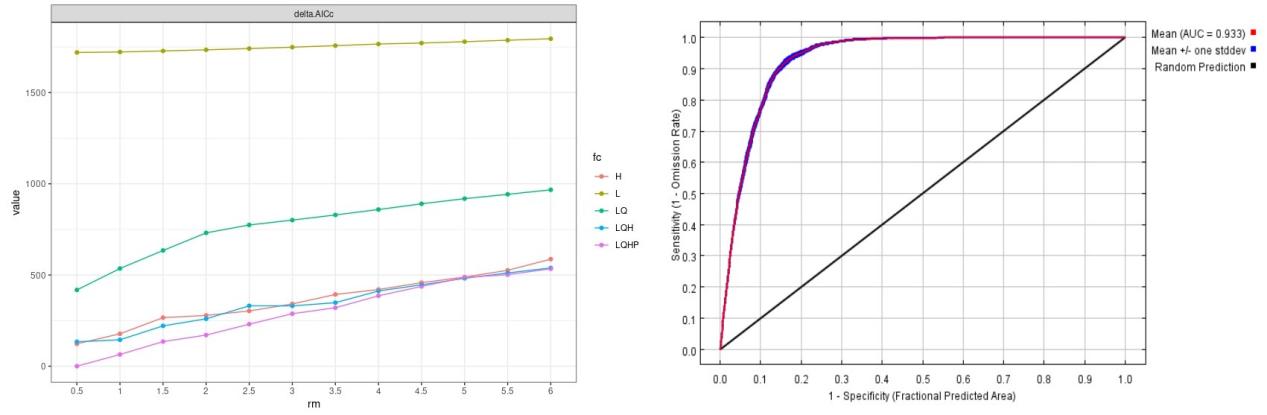


Fig. S1 Optimal parameter combinations (left) and mean AUC values (right) for the optimised MaxEnt model (L: linear; Q: quadratic; H: hinge; P: product; T: threshold; AUC: the area under receiver operating characteristic (ROC) curve)
